# Exploring the Nexus of lower extremity proprioception and postural stability in older adults with osteoporosis: a cross-sectional investigation

**DOI:** 10.3389/fpubh.2023.1287223

**Published:** 2023-11-30

**Authors:** Irshad Ahmad, Ravi Shankar Reddy, Raee S. Alqhtani, Jaya Shanker Tedla, Snehil Dixit, Hussain Saleh H. Ghulam, Abdullah Mohammed Alyami, Saeed Al Adal, Mohammad A. M. Jarrar

**Affiliations:** ^1^Program of Physical Therapy, Department of Medical Rehabilitation Sciences, College of Applied Medical Sciences, King Khalid University, Abha, Saudi Arabia; ^2^Physical Therapy Department Medical Applied Sciences College, Najran University, Najran, Saudi Arabia; ^3^Rehabilitation Sciences Department, Applied Medical Sciences College, Najran University, Najran, Saudi Arabia

**Keywords:** lower extremity proprioception, postural stability, osteoporosis, center of pressure displacement, sway velocity

## Abstract

**Background:**

Osteoporosis, characterized by reduced bone mass and micro-architectural deterioration, poses a significant public health concern due to increased fracture susceptibility. Beyond bone health, this cross-sectional study aimed to assess and compare lower extremity proprioception and postural stability in individuals with and without osteoporosis and to explore their correlation within the osteoporosis group.

**Method:**

In this prospective cross-sectional study, 80 participants were divided into two groups: osteoporosis (*n* = 40) and control (*n* = 40). The demographic characteristics and clinical parameters of the participants were as follows: Age (years) – Osteoporosis group: 65.04 ± 4.33, Control group: 65.24 ± 4.63; Sex (%) – Osteoporosis group: Male 30%, Female 70%; Control group: Male 30%, Female 70%; Body mass index (kg/m^2^) – Osteoporosis group: 23.7 ± 3.2, Control group: 24.5 ± 4.6; T-score (Lumbar) – Osteoporosis group: −2.86 ± 1.23, Control group: 0.27 ± 0.58; T-score (hip) – Osteoporosis group: −2.28 ± 0.79, Control group: 0.68 ± 0.86. Joint Position Sense (JPS) at the hip, knee, and ankle was assessed using a digital inclinometer, and postural stability was measured using computerized force platforms.

**Result:**

Osteoporosis participants exhibited higher errors in hip (5.63° vs. 2.36°), knee (4.86° vs. 1.98°), and ankle (4.46° vs. 2.02°) JPS compared to controls. Postural stability measures showed increased anterior–posterior sway (10.86 mm vs. 3.98 mm), medial-lateral sway (8.67 mm vs. 2.89 mm), and ellipse area (966.88 mm^2^ vs. 446.19 mm^2^) in osteoporosis participants. Furthermore, correlation analyses within the osteoporosis group unveiled significant positive associations between lower extremity proprioception and postural stability. Specifically, hip JPS exhibited a strong positive correlation with anterior–posterior sway (*r* = 0.493, *p* = 0.003), medial-lateral sway (*r* = 0.485, p = 0.003), and ellipse area (*r* = 0.496, *p* < 0.001). Knee JPS displayed a moderate positive correlation with anterior–posterior sway (*r* = 0.397, *p* = 0.012), medial-lateral sway (*r* = 0.337, *p* = 0.032), and ellipse area (*r* = 0.378, *p* < 0.001). Similarly, ankle JPS showed a moderate positive correlation with anterior–posterior sway (*r* = 0.373, *p* = 0.023), medial-lateral sway (*r* = 0.308, *p* = 0.045), and ellipse area (*r* = 0.368, *p* = 0.021).

**Conclusion:**

These findings underscore the interplay between proprioceptive deficits, compromised postural stability, and osteoporosis, emphasizing the need for targeted interventions to improve fall prevention strategies and enhance the quality of life for individuals with osteoporosis.

## 1 Introduction

Osteoporosis is a systemic skeletal disorder characterized by low bone mass and micro-architectural deterioration, leading to increased bone fragility and susceptibility to fractures (1). It is a major public health concern affecting millions worldwide, particularly postmenopausal women and the older adults (2). Osteoporotic fractures can result in significant morbidity, mortality, and reduced quality of life (3). While bone health is a primary focus in osteoporosis management, it is also important to consider other factors that contribute to the risk of falls and fractures, such as proprioception, postural stability, and gait stability (4, 5).

Proprioception, the perception and awareness of limb and body position, is critical for motor control and maintaining balance during functional activities (6). It relies on a complex interplay of sensory inputs from mechanoreceptors in muscles, tendons, and joints (6). Proprioceptive deficits have been observed in musculoskeletal and neuromuscular conditions, contributing to impaired motor control, decreased postural stability, and an increased risk of falls (7). Understanding the role of proprioception in individuals with osteoporosis is crucial as it may have implications for fall prevention strategies and interventions.

Postural stability is a fundamental aspect of maintaining an upright stance and coordinating bodily movements (8). It relies on the integration of sensory information from various systems, including the visual, vestibular, and somatosensory systems (9). These sensory systems collaborate to provide the necessary inputs for the body to adapt its posture and sustain balance (10). The visual system supplies information about the surrounding environment, the vestibular system contributes data regarding head movements and spatial orientation, and the somatosensory system conveys feedback from muscles and joints (11). Disruptions in this delicate equilibrium can significantly elevate the risk of stumbling and subsequent fractures, particularly among individuals with osteoporosis (12). Investigating postural stability not only offers insights into the consequences of proprioceptive deficits but also identifies individuals at an increased risk of balance-related incidents (13). A comprehensive understanding of the mechanisms governing postural stability is essential, as it illuminates its crucial role in everyday activities and underscores its significance in the context of osteoporosis and proprioception.

Bone mineral density (BMD) is a fundamental measure of bone health, reflecting the mineral content, primarily calcium and phosphorus, in bone tissue, and serving as a crucial indicator of bone strength and density (14). In our study, BMD was assessed as part of the inclusion criteria to ensure that participants in the osteoporosis group met the diagnostic criteria for osteoporosis based on established BMD thresholds (reference citation). Low BMD, a hallmark of osteoporosis, can exert a substantial influence on lower extremity proprioception and balance through a multifaceted mechanism (15). Reduced bone density can lead to structural changes in the skeletal system, including vertebral deformities and diminished bone strength (16). These skeletal alterations can disrupt the alignment of the spine, impacting the proprioceptive feedback loop, as mechanoreceptors within the spine play a critical role in conveying information about body position (17). Consequently, individuals with low BMD may experience a diminished ability to accurately perceive the orientation of their lower extremities and trunk, potentially impairing their proprioceptive acuity (18). Moreover, diminished bone density is associated with an elevated risk of fragility fractures, which can cause physical pain, and functional limitations, and instill fear of falling (18). This fear can lead to altered movement patterns and heightened muscle tension, further perturbing proprioceptive input (19). Additionally, fractures can result in chronic pain and muscle weakness, negatively impacting postural stability and balance (20, 21). Collectively, low BMD can disrupt the intricate interplay between skeletal integrity, sensory input, and neuromuscular control, ultimately predisposing individuals to proprioceptive deficits and impaired balance, which are critical factors contributing to the heightened fall risk observed in osteoporotic individuals (22).

This study aims to address a significant research gap by investigating the intricate relationships between osteoporosis, proprioceptive function, and postural stability. Osteoporosis, characterized by reduced bone mass and micro-architectural deterioration, poses a substantial public health concern due to the increased susceptibility to fractures. While the impact of osteoporosis on bone health is well-established, there is a limited understanding of how it affects proprioceptive function and, consequently, postural stability. Proprioception, the ability to perceive the position and movement of one’s body in space, plays a critical role in postural control. However, the association between proprioceptive deficits and compromised postural stability in individuals with osteoporosis remains underexplored. The research aims to achieve a more comprehensive understanding of these interrelated factors by conducting a thorough assessment and comparison. Specifically, we focus on evaluating lower extremity proprioception using established methods such as joint position sense tests, kinesthetic sense evaluations, and passive motion measurements. In parallel, we employ rigorous quantitative measures to assess postural stability, including the center of pressure displacement, sway velocity, and postural control indices derived from force platforms. The primary research aims are as follows: (1) To assess and compare lower extremity proprioception and postural stability between individuals with osteoporosis and a control group without osteoporosis. (2) To explore the correlations between lower extremity proprioception and postural stability within the osteoporosis group.

Our research hypothesis posits that individuals with osteoporosis will demonstrate markedly impaired proprioceptive function and diminished postural stability in comparison to the control group. Furthermore, we anticipate observing substantial negative correlations between proprioceptive function and measures of postural stability within the osteoporosis group, indicative of a strong association between greater proprioceptive deficits and reduced postural stability. By shedding light on these associations and identifying specific proprioceptive impairments contributing to postural instability in individuals with osteoporosis, this study seeks to pave the way for the development of targeted interventions. These interventions aim to enhance proprioceptive function, improve postural control, and ultimately reduce the risk of falls and fractures within this vulnerable population.

## 2 Materials and methods

### 2.1 Participants

In this prospective cross-sectional study, 80 participants were divided into two groups: osteoporosis (*n* = 40) and control (*n* = 40) (Table 1). The demographic characteristics and clinical parameters of the participants were as follows: Age (years) – Osteoporosis group: 65.04 ± 4.33, Control group: 65.24 ± 4.63; Sex (%) – Osteoporosis group: Male 30%, Female 70%; Control group: Male 30%, Female 70%; Body mass index (kg/m^2^) – Osteoporosis group: 23.7 ± 3.2, Control group: 24.5 ± 4.6; T-score (Lumbar) – Osteoporosis group: −2.86 ± 1.23, Control group: 0.27 ± 0.58; T-score (hip) – Osteoporosis group: −2.28 ± 0.79, Control group: 0.68 ± 0.86. Joint Position Sense (JPS) at the hip, knee, and ankle was assessed using a digital inclinometer, and postural stability was measured using computerized force platforms. The groups exhibited no significant differences in age, sex distribution, weight, height, or body mass index. However, individuals with osteoporosis had significantly lower T-scores at both lumbar and hip regions compared to healthy individuals, indicating lower bone density in the osteoporosis group. Participants for this study were recruited from diverse sources to ensure the representativeness of the sample, taking into consideration eligibility criteria, which included both inclusion and exclusion parameters. A substantial portion of our cohort was enrolled through the rheumatology outpatient clinic at KKU Hospital, where individuals with known or suspected osteoporosis were approached during their clinic visits. Additionally, we extended our recruitment efforts by advertising the study in various medical clinics within the Abha, Asser region, thereby reaching a broader population of potential participants. Collaborations were also established with clinics specializing in densitometric measurements, facilitating the inclusion of patients seeking osteoporosis-related assessments. In a bid to capture a wide spectrum of participants, including those who might not typically seek medical care, recruitment efforts were conducted in urban entertainment districts, engaging individuals from the general public.

**Table 1:** Baseline participant characteristics.

Variables	Osteoporosis individuals (*n* = 40)	Healthy individuals (*n* = 40)
Age (years)	65.04 ± 4.33	65.24 ± 4.63
Sex: *n* (%)		
Male	12 (30)	12 (30)
Female	28 (70)	28 (70)
Weight (kg)	72.63 ± 5.96	70.46 ± 5.32
Height (cm)	164.9 ± 7.45	161.65 ± 5.43
Body mass index (kg/m^2^)	23.7 ± 3.2	24.5 ± 4.6
T-score (Lumbar)	−2.86 ± 1.23	0.27 ± 0.58
T-score (hip)	−2.28 ± 0.79	0.68 ± 0.86

### 2.2 Eligibility criteria

The criteria for inclusion in this study were intentionally stringent to ensure specificity. Individuals underwent thorough screening of their medical histories and were excluded if they had a past or present diagnosis of conditions such as malignancy, chronic hepatitis, renal ailments, persistent gastrointestinal disorders, rheumatoid arthritis, parathyroid anomalies, hyperthyroidism, or diabetes mellitus. Furthermore, men who had experienced vertebral fractures in the preceding 6 months, those with incapacitating conditions impairing their ability to carry out daily activities autonomously, or those grappling with intense and chronic back pain that could potentially interfere with assessments of proprioception and balance were also intentionally excluded. The study further excluded individuals who had taken bisphosphonates or vitamin D supplements within the last year, seeking to isolate a cohort that had not been influenced by these factors.

Conversely, the study encompassed individuals of sound health, devoid of any history of osteoporosis, fractures arising from mild trauma, substantial bone-related disorders, or medications that could impact bone health. The participants were also required to willingly provide informed consent and display bone density measurements that fell within the normal range (T-score > −1.0). Individuals who were pregnant or breastfeeding were earmarked for exclusion. These comprehensive criteria served as the foundation for both research and clinical investigations, with potential variations considered based on the specific parameters of each study.

### 2.3 Study design

Conducted within the settings of orthopedic clinics – at King Khalid University, this prospective cross-sectional study aimed to comprehensively evaluate and juxtapose lower extremity proprioception and postural stability in individuals with and without osteoporosis. Ethical approval was obtained from the University Ethics Board (KKU, [REC# 230-34-879]) before the initiation of data collection. Conforming to the principles established in the Declaration of Helsinki, this study meticulously followed ethical guidelines and standards throughout its design, execution, and analysis. The study spanned from April 2020 to December 2022, comprehensively examining the objectives in older adults individuals above 60.

### 2.4 Sample size calculation

The sample size for this study was determined based on statistical considerations to ensure adequate power to detect significant differences in lower extremity proprioception and postural stability between the osteoporosis group and the control group. The effect size (0.6) used for this calculation was derived from a previous study by Cuaya-Simbro et al. (23) which investigated similar outcome measures in a comparable population. Using a power of 0.80 and a significance level of 0.05, the analysis indicated that a minimum sample size of 40 subjects in each group would provide sufficient statistical power to detect meaningful differences. Therefore, a total of 80 participants were recruited, with 40 individuals in the osteoporosis group and 40 in the control group.

### 2.5 Lower extremity proprioception assessment

Joint position sense (JPS) assessment is a fundamental method for evaluating an individual’s ability to perceive and replicate specific joint angles accurately (24). This study applied the assessment to the hip, knee, and ankle joints, each employing distinct protocols and methodologies to evaluate proprioceptive abilities comprehensively. The assessments were meticulously conducted within a controlled and serene environment to minimize potential external factors that could influence participants’ concentration and proprioceptive acuity (25). The objective was to ensure consistent testing conditions across all participants. To uphold standardization, the same set of equipment, “digital inclinometers,” (J-Tech Medical, Midvale, UT, United States) was utilized for assessing all three joint positions. Skilled and trained examiners well-versed in musculoskeletal assessment and proprioception were entrusted with administering the evaluations. Their expertise contributed to the precision of inclinometer placement, precise guidance through joint movements, and overall reliability of the measurement process. A crucial aspect of the assessment was the implementation of blindfolding during the proprioception testing. Blindfolding participants effectively eliminated visual input, ensuring that proprioceptive cues were the primary sensory modality employed during the JPS assessments.

A significant methodology adopted throughout the assessments was the patient active repositioning technique (26). This technique required participants to actively reposition their joints to match a target angle after being guided to that angle by the examiner (27). To accurately quantify joint angles, digital inclinometers were employed. These advanced instruments facilitated precise and consistent measurements of joint angles throughout the testing procedures, thereby enhancing the reliability and validity of the outcomes.

#### 2.5.1 Hip joint position sense assessment

**Figure 1:**
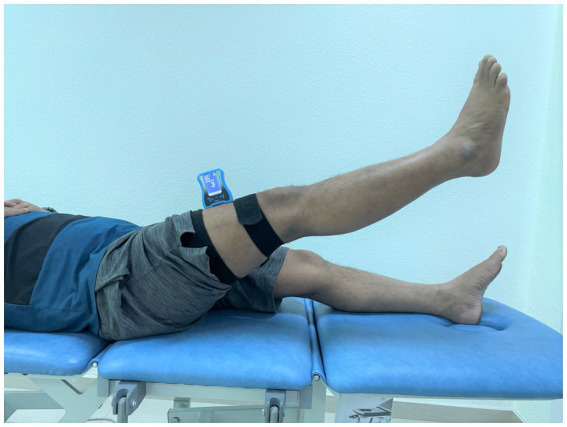
Assessment of hip joint position sense utilizing a dual digital inclinometer.

The evaluation of hip joint position sense concentrated on realigning the hip to a 60-degree flexion angle while lying supine (28). For the assessment of hip JPS in flexion, a digital inclinometer was positioned on the front and center of the individual’s thigh. It was firmly secured in place using a hook-and-loop strap (Figure 1). The evaluated limb was brought to a 60-degree flexion angle during the test (28). The examiner held the participant’s limb steady at this 60-degree flexion angle, which served as the designated target position (28). This position was sustained for 5 s, allowing the participant to remember the sensation. Subsequently, the participant’s hip was guided back to the initial starting position. After this, the participants were responsible for realigning their hips to the target position. This successful realignment was indicated by the participant affirming with a “Yes” once they felt they had accurately matched the target angle. Ultimately, the measure of hip joint position sense accuracy involved calculating the absolute difference, in degrees, between the initially established target angle and the angle successfully reproduced by the participant. This difference, the joint position error, was the metric for evaluating JPS accuracy.

#### 2.5.2 Knee joint position sense assessment

**Figure 2:**
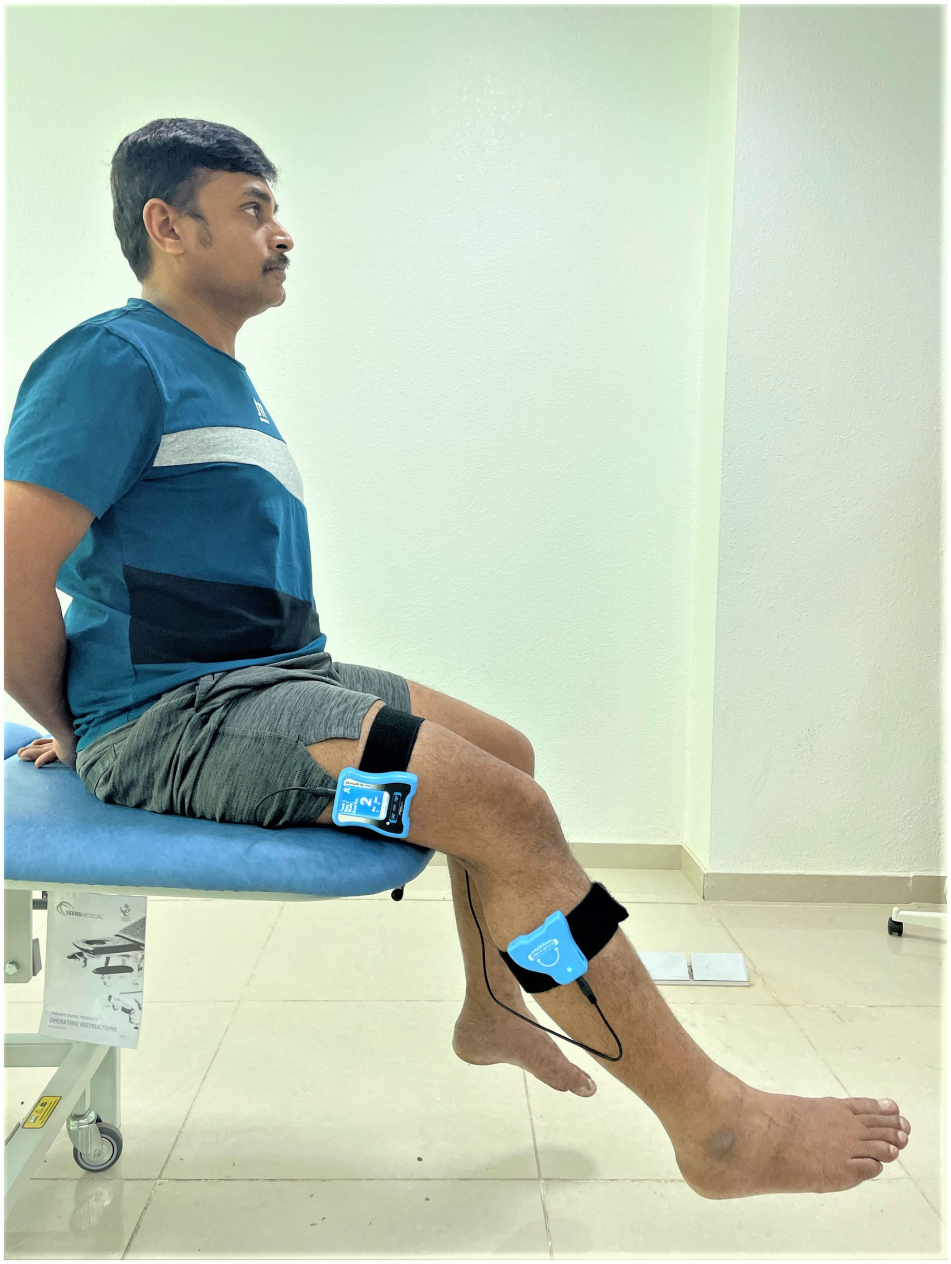
Assessment of knee joint position sense utilizing a dual digital inclinometer.

Knee JPS was assessed in a sitting position, with the subjects being asked to reposition the knee to a 45-degree angle actively (29). Participants were seated comfortably on a chair, with an inclinometer securely fastened to their knee joint area. Specifically, one part of the inclinometer was attached to the lower one-third section of the femur’s lateral surface along the joint line (30). In contrast, the other part was attached to the lower leg’s lateral segment along the joint line (Figure 2). To carry out the measurement, the examiner initiated the process from a starting point where the knee was flexed at a 90-degree angle. From there, the examiner guided the participant’s knee to achieve the target angle of 45 degrees (30). This target angle was held for 5 s. Subsequently, participants were asked to remember this specific target position mentally. Following this, the knee was guided back to the initial starting position. At this point, participants were instructed to reproduce the remembered target knee angle accurately. This successful realignment was indicated by the participant affirming with a “Yes” once they felt they had accurately matched the target angle. The level of accuracy in reproducing this angle was then evaluated in degrees, providing insight into the participants’ knee joint position sense.

#### 2.5.3 Ankle joint position sense assessment

**Figure 3:**
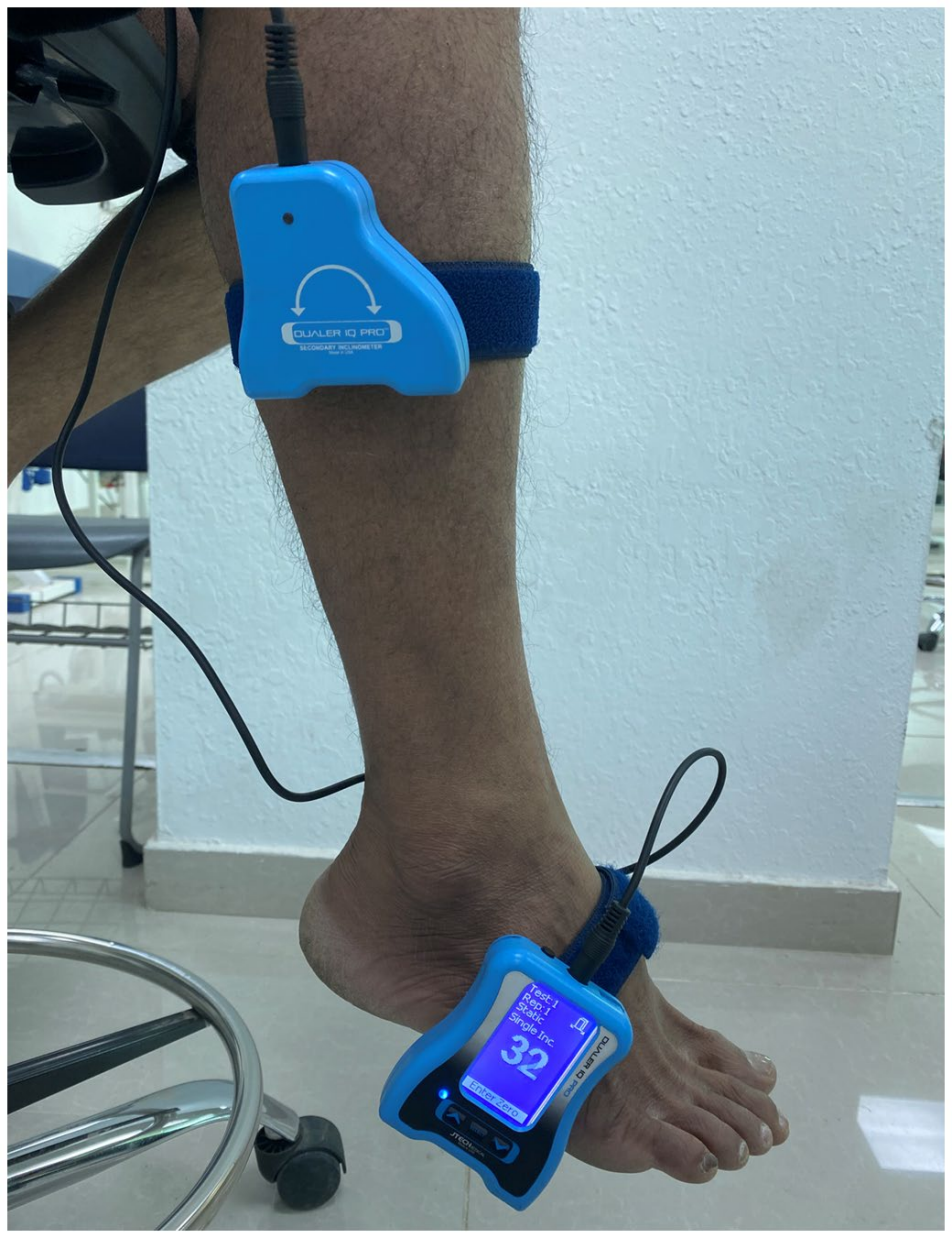
Assessment of ankle joint position sense utilizing a dual digital inclinometer.

Volunteers in the study underwent a precise procedure involving the adjustment of their foot placement to attain a specific angle of 15 degrees of plantar flexion (31). For this process, the participants were directed to take a seat on a couch with their eyes shut, adopting an elevated sitting posture. Employing a Velcro strap, one component of a dual inclinometer was securely fastened to the lateral side’s midsection of their tibia, while the primary inclinometer was affixed to the outer edge of the foot (Figure 3) (32). With the examiner’s guidance, the participant’s foot was manipulated to achieve the designated 15-degree plantarflexion angle, which was then sustained for 5 s. During this interval, participants were instructed to mentally register and remember this particular foot position. Subsequently, the foot was guided back to a neutral or initial position. At this juncture, participants were tasked with actively repositioning their ankles to replicate the aforementioned target angle (32). Upon successfully reaching and confidently matching the intended position, they signaled their accomplishment by verbally affirming “YES.” This sequence of steps formed a meticulous protocol employed to assess the participants’ ability to both achieve and recall the specific 15-degree plantarflexion angle. The JPS evaluations were carried out through a sequence of three successive trials for each examined direction, meticulously undertaken to guarantee precision. The resulting mean value from this trio of trials was subsequently utilized for subsequent analytical procedures. The directives provided to the examiners remained uniform and consistent across all of these testing instances.

### 2.6 Postural stability assessment

**Figure 4:**
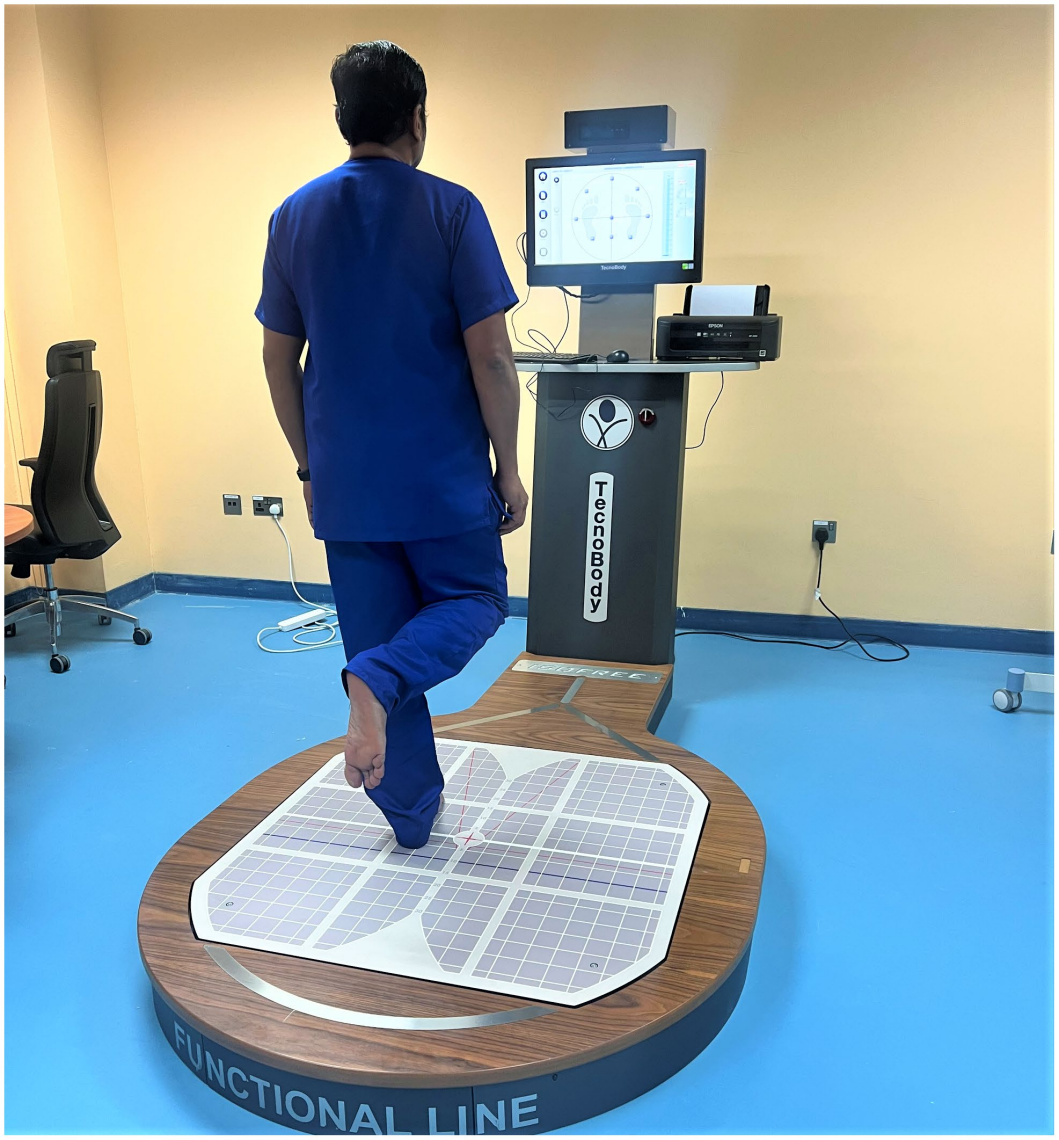
Computerized IsoFree stabilometric force platform for quantitative postural stability assessment in the study participants.

The evaluation of postural stability was conducted by employing a stabilometric force platform, (IsoFree medical equipment, Tecnobody, SRL, Dalmine (BG) – Italy) which constituted the foundational cornerstone of the assessment procedure (32, 33). The commencement of this rigorous evaluation encompassed the precise calibration of the force platform to uphold the standards of accuracy (34). Study participants stood upon the force platform of the posturography device, wearing appropriate attire such as spandex shorts or a garment with similar tactile properties. During this evaluation, participants were guided to adopt a single-leg stance, with the contralateral leg flexed away from the force platform while allowing their hands to rest naturally at their sides (34). In this stance, participants were directed to focus on a designated target marker displayed on a computer monitor (Figure 4). The participants were tasked with sustaining this one-legged stance for 30 s, all while keeping their gaze fixed upon the “X” target mark. Furthermore, they were advised to ensure that their arms remained relaxed and uninvolved during the evaluation to maintain consistency. In each testing instance, individuals were required to uphold this stance for 30 s. This protocol was repeated twice, resulting in two trials, and subsequently, the trial exhibiting the most favorable performance was chosen for further analysis (35). Noteworthy parameters related to postural stability (A-P sway in mm, M-L sway in mm, and Ellipse area in mm^2^) were meticulously quantified during these trials.

In this study, lower extremity proprioception assessment and postural stability assessment were exclusively performed in the dominant legs of the participants. This approach was chosen to focus on the specific proprioceptive capabilities and postural stability of the dominant leg, which is typically more actively engaged in daily activities and often plays a crucial role in maintaining balance during various tasks.

Table 2 outlines the controlled variables and their respective categories (dependent, independent, measuring, and confounding).

**Table 2:** Summary of controlled variables.

Variable	Category
Dependent Variables	
Lower Extremity Proprioception Measures	
Hip JPS	Measuring
Knee JPS	Measuring
Ankle JPS	Measuring
Postural stability measures	
A-P Sway	Measuring
M-L Sway	Measuring
Ellipse Area	Measuring
Independent Variables	
Group (Osteoporosis vs. Control)	
Dominant Leg	
Measuring Variables	
Age	Confounding
Physical Activity	Confounding
Bone Mineral Density	Confounding
BMI	Confounding

JPS, joint position sense; A-P, Anterior–Posterior; M-L, Medial-Lateral; BMI, body mass index.

### 2.7 Data analysis

The data analysis phase of this study relied on parametric statistical methods, as the data exhibited a normal distribution as confirmed by the Shapiro–Wilk test (36). The analyses were conducted using SPSS version 20 (IBM Corp., Armonk, NY, United States). To assess statistical significance, a predefined threshold of *p* < 0.05 was selected, ensuring a robust evaluation of the research objectives. To compare key variables such as lower extremity proprioception and postural stability between individuals with osteoporosis and those without, independent *t*-tests were applied. Effect sizes, specifically Cohen’s *d*, were calculated to gauge the magnitude of differences between these groups. Effect sizes were interpreted following Cohen’s established criteria, categorizing them as small (*d* = 0.2), medium (*d* = 0.5), or large (*d* = 0.8) (37). Furthermore, to delve into the relationship between lower extremity proprioception and postural stability, particularly among individuals with osteoporosis, Pearson’s correlation coefficient (*r*) was employed. The strength of these correlations was characterized as trivial (0.00–0.10), small (0.10–0.30), moderate (0.30–0.50), large (0.50–0.70), very large (0.70–0.90), or nearly perfect (0.90–1.00) based on widely recognized standards (38). In addition to hypothesis testing, descriptive statistics played a pivotal role in elucidating the study’s findings. Continuous variables were presented as Mean ± Standard Deviation (M ± SD), offering a clear representation of central tendencies and variability. Categorical data were summarized using frequencies and percentages, providing a comprehensive overview of the sample characteristics. Moreover, mean or percentage differences between groups for relevant outcome measures, along with their corresponding 95% confidence intervals, were calculated and reported, facilitating a deeper understanding of the practical significance of observed differences.

## 3 Results

Table 2 summarizes the baseline characteristics of participants, dividing them into osteoporosis individuals (*n* = 40) and healthy individuals (*n* = 40). The groups exhibited no significant differences in age, sex distribution, weight, height, or body mass index. However, individuals with osteoporosis had significantly lower T-scores at both lumbar and hip regions compared to healthy individuals, indicating lower bone density in the osteoporosis group (see Table 3).

**Table 3:** Lower extremity joint position errors and postural stability measures between osteoporosis and healthy participants.

Variables	Osteoporosis participants (*n* = 40)	Healthy controls (*n* = 40)	Mean difference (95% CI)	Percentage difference (95% CI)	Effect size (Cohen’s *d*)
Hip JPS (°) Knee JPS (°) Ankle (°)	5.63 ± 1.83 4.86 ± 1.96 4.46 ± 2.01	2.36 ± 0.86 1.98 ± 0.67 2.02 ± 1.32	0.83 (0.56) 0.38 (0.53) 0.21 (0.46)	22.36 (11.6, 31.09) 21.24 (08.36, 28.36) 20.44 (07.73, 16.86)	0.95 (0.43, 1.41) 0.89 (0.33, 1.40) 0.76 (0.23, 1.38)
Postural stability variables Anterior–posterior sway (mm) Medial-lateral sway (mm) Ellipse area (mm^2^)	10.86 ± 3.017 8.67 ± 2.26 966.88 ± 158.56	3.98 ± 1.12 2.89 ± 1.34 446.19 ± 135.48	1.96 (0.23, 1.43) 2.34 (1.02, 2.54) 235.35 (198.98, 412.67)	22.6 (9.9, 31.6) 19.7 (11.6, 34.9) 32.6 (15.8, 39.6)	3.26 (1.53, 4.98) 3.89 (1.33, 4.40) 4.95 (2.43, 5.41)

JPS, joint position sense; CI, confidence interval.

In the comparison between osteoporosis participants and healthy controls (Table 2), osteoporosis participants exhibited significantly higher joint position errors (JPS) in hip, knee, and ankle positions compared to healthy controls. These differences were statistically significant, with osteoporosis participants showing increases of 0.83° for hip JPS, 0.38° for knee JPS, and 0.21° for ankle JPS. Additionally, regarding postural stability variables, osteoporosis participants displayed significantly increased anterior–posterior sway, medial-lateral sway, and ellipse area compared to healthy controls. These differences were also statistically significant, with osteoporosis participants demonstrating greater sway in anterior–posterior (1.96 mm), medial-lateral (2.34 mm), and a larger ellipse area (235.35 mm^2^). Effect sizes for these postural stability measures were notably substantial, with values of 3.26 for anterior–posterior sway, 3.89 for medial-lateral sway, and 4.95 for the ellipse area, emphasizing the significant differences in postural stability between the two groups.”

In individuals with osteoporosis, a correlation analysis (Table 4) revealed significant positive correlations. Hip JPS correlated positively and moderately with anterior–posterior sway (*r* = 0.493, *p* = 0.003), medial-lateral sway (*r* = 0.485, *p* = 0.003), and ellipse area (*r* = 0.496, *p* < 0.001). Knee JPS also showed a positive correlation, albeit of moderate strength, with anterior–posterior sway (*r* = 0.397, *p* = 0.012), medial-lateral sway (*r* = 0.337, *p* = 0.032), and ellipse area (*r* = 0.378, *p* < 0.001). Ankle JPS exhibited positive correlations, with moderate strength, anterior–posterior sway (*r* = 0.373, *p* = 0.023), medial-lateral sway (*r* = 0.308, *p* = 0.045), and ellipse area (*r* = 0.368, *p* = 0.021).

**Table 4:** Correlation between lower extremity joint position errors and postural stability measures in individuals with osteoporosis.

Variables		Anterior–posterior sway (mm)	Medial-lateral sway (mm)	Ellipse area (mm^2^)
Hip JPS (°)	*r*	0.493	0.485	0.496
	Value of *p*	0.003	0.003	<0.001
Knee JPS (°)	*r*	0.397	0.337	0.378
	Value of *p*	0.012	0.032	<0.001
Ankle (°)	*r*	0.373	0.308	0.368
	Value of *p*	0.023	0.045	0.021

JPS, joint position sense.

## 4 Discussion

In this study, we set out to achieve two primary objectives. First, we aimed to comprehensively evaluate and compare lower extremity proprioception and postural stability between individuals with osteoporosis and those without the condition. Secondly, we sought to investigate the complex relationship between lower extremity proprioception and postural stability specifically within the group of individuals with osteoporosis. We hypothesized that individuals with osteoporosis would exhibit significantly impaired proprioceptive function and compromised postural stability compared to the control group. Additionally, we posited that there would be significant negative correlations between proprioceptive function and postural stability measures within the osteoporosis group, indicating that greater proprioceptive deficits are associated with reduced postural stability. We can confirm that our study results are in line with these hypotheses, as we observed that individuals with osteoporosis indeed displayed significantly impaired proprioceptive function and compromised postural stability when compared to the control group. Furthermore, we identified significant negative correlations between proprioceptive function and postural stability measures within the osteoporosis group, providing support for our initial hypotheses.

Regarding JPS, individuals with osteoporosis were found to manifest considerably higher errors in hip, knee, and ankle positions in contrast to their healthy counterparts. Compared to healthy controls, the diminished lower extremity proprioception observed in individuals with osteoporosis can be attributed to a combination of factors. Various factors contribute to the decline in proprioception in individuals with osteoporosis (39). These factors encompass reduced bone mass and changes in microarchitecture, which disrupt proprioceptive receptors in bones and joints (40). Additionally, muscle weakness and imbalances related to osteoporosis affect muscle spindle feedback and the stretch reflex (41). Joint degeneration alters mechanoreceptor distribution and kinematics, while vertebral fractures can lead to potential neuropathy and nerve compression, impacting sensory signal transmission (42). Impaired blood flow can compromise the health and function of receptors, and psychological factors may influence movement patterns and sensory perception (43). Furthermore, age-related changes in sensory receptors and central processing can exacerbate proprioceptive decline (44–47). This intricate interplay of physiological, biomechanical, and structural elements collectively contributes to the observed proprioceptive deficits, underscoring the complexity of osteoporosis’s impact on sensory function and motor control (47, 48). The observed statistically significant mean differences, percentage disparities, and effect sizes underscore these findings’ clinical relevance and importance, emphasizing their potential ramifications on balance and coordination for individuals with osteoporosis.

The realm of postural stability variables further accentuated the dissimilarity between the two groups. Participants with osteoporosis exhibited considerably heightened anterior–posterior sway, medial-lateral sway, and ellipse area compared to their healthy counterparts. The statistically significant mean differences elucidate the evident instability among osteoporosis participants. The compromised postural stability observed in individuals with osteoporosis compared to healthy controls can be attributed to a range of interconnected factors (49, 50). Several factors contribute to the observed instability in individuals with osteoporosis. These factors encompass altered bone structure, which weakens skeletal integrity, and muscle weakness and imbalances that hinder dynamic stability (51). Additionally, impaired joint function and limited range of motion play a role, as do diminished proprioception, affecting the body’s ability to sense and respond to postural changes (52, 53). Degenerative changes in spinal alignment impact the center of mass distribution, while impaired neuromuscular coordination and altered muscle recruitment patterns further exacerbate the issue (54). There are also potential sensory alterations impacting the integration of sensory information, and fear of falling can influence movement behavior (55, 56). Medication effects can worsen muscle weakness and bone fragility, and age-related declines in muscle mass, strength, and sensory perception collectively contribute to the observed instability (16, 57–59). The research studies encompassed in the provided summaries shed light on the amplified postural instability observed in participants with osteoporosis when compared to their healthy counterparts. The study, conducted by Okayama et al., (49) examined postmenopausal women with osteoporosis and revealed a higher prevalence of sarcopenia, which correlated with reduced quality of life scores, increased postural instability, and a greater history of falls (27). This underscores the intricate relationship between musculoskeletal health and postural control. The study by Simon et al. (50) investigated a larger cohort and demonstrated that lower femoral bone mineral density (BMD) T-scores, along with factors like age and sex, were associated with heightened path length during Romberg posturography, signifying compromised postural stability. The study by Rezaei (60) delved into dynamic postural control and found that participants with type-I osteoporosis exhibited reduced sway velocity and excursion during weight shifting and dynamic tasks, indicating the influence of bone mineral density decline on these aspects of postural control. Together, these studies emphasize the multidimensional impact of osteoporosis on postural stability, providing valuable insights for clinical interventions and fracture prevention strategies. This multifaceted interplay underscores the need for comprehensive interventions addressing bone health and motor control to enhance postural stability and minimize fall risk in individuals with osteoporosis.

Limited studies have comprehensively explored the objectives of evaluating lower extremity proprioception and postural stability in individuals with osteoporosis and their interplay, underscoring the significance of our research. To encapsulate, the findings from this investigation underscore marked deficits in joint position sense and postural stability among individuals with osteoporosis when contrasted with healthy controls. The correlation between lower extremity proprioceptive loss and impaired postural stability in individuals with osteoporosis can be attributed to a complex interplay of factors. Compromised proprioception disrupts accurate sensory input, leading to altered movement strategies and impaired anticipatory adjustments (61, 62). This results in delayed feedback loops, compromised joint stabilization, and reduced movement variability, ultimately undermining the body’s ability to respond to postural challenges efficiently (63). Muscular inefficiencies arise from inadequate muscle activation patterns, while impaired joint sensation and decreased feedforward mechanisms hinder the body’s ability to sense joint positions and pre-activate muscles for stability (64). For instance, Ucurum et al. (59) reported analogous disruptions in joint position sense and amplified postural sway in a cohort of osteoporosis patients. Collectively, these mechanisms contribute to the observed correlation, emphasizing the critical role of proprioception in maintaining postural control and highlighting the need for interventions addressing both proprioceptive deficits and broader motor control aspects to enhance postural stability in osteoporosis.

The clinical significance of these findings is their potential to inform targeted interventions for mitigating postural instability and fall risk in individuals with osteoporosis (65). Understanding the intricate relationship between lower extremity proprioceptive loss and impaired postural stability offers insights into the multifaceted nature of motor control deficits in this population (66). Healthcare professionals can develop tailored rehabilitation strategies that address bone health, enhance proprioception through sensory training, tackle muscular imbalances, and promote optimal movement patterns (66, 67). By addressing both bone health and broader motor control aspects, interventions may reduce falls, fractures, and associated morbidity, ultimately improving the quality of life and functional independence of individuals with osteoporosis (68). Future research avenues could explore neurophysiological mechanisms, longitudinal progression of deficits, innovative interventions, and moderating factors to deepen our understanding and guide personalized interventions (69).

The findings from this study have several practical implications for healthcare professionals and researchers working with individuals with osteoporosis. First and foremost, our results confirm that individuals with osteoporosis exhibit significantly impaired proprioceptive function and compromised postural stability compared to those without the condition (50). This underscores the importance of routine assessments of proprioception and postural stability in clinical settings for individuals with osteoporosis (70). Healthcare providers can use these assessments to identify individuals at higher risk of falls and fractures, allowing for targeted interventions to enhance proprioceptive function and mitigate fall risk (71). Moreover, the significant negative correlations between proprioceptive function and postural stability measures within the osteoporosis group highlight the interplay between these factors. Addressing both proprioceptive deficits and broader motor control aspects is essential for improving postural stability in individuals with osteoporosis (72). Healthcare professionals can design tailored rehabilitation strategies that not only focus on bone health but also incorporate sensory training, address muscular imbalances, and promote optimal movement patterns (73). These interventions may help reduce falls, fractures, and associated morbidity, ultimately enhancing the quality of life and functional independence of individuals with osteoporosis (74). Future research in this field should explore neurophysiological mechanisms, the longitudinal progression of deficits, innovative interventions, and moderating factors to deepen our understanding and guide personalized interventions (74). By addressing these areas, researchers and clinicians can continue to advance our knowledge and develop more effective strategies for preventing falls and fractures in individuals with osteoporosis.

While this study contributes valuable insights into the relationships among proprioception, postural stability, and osteoporosis, it is important to acknowledge its limitations. The study’s cross-sectional design precludes the establishment of causal relationships between these factors, and longitudinal studies would be better suited to investigate their temporal dynamics and predictive value regarding fall risk. Moreover, the sample size of 40 participants, while meticulously calculated and recruited, may restrict the generalizability of the findings. Although larger samples can reduce associated error, amplification procedures such as bootstrapping techniques offer opportunities for greater expansion of inferences to the study’s target population. Expanding and diversifying the participant pool in future research could further enhance external validity. Additionally, while validated methods were used to assess lower extremity proprioception and postural stability, the study did not comprehensively address other contributors to fall risk, such as muscle strength, cognitive function, and fear of falling. Future research could explore these multifaceted aspects to gain a more holistic understanding of fall risk in individuals with osteoporosis. Another limitation is the omission of a comprehensive assessment of potential comorbidities, such as sarcopenia, which can influence proprioception and postural stability in older adults with osteoporosis. Incorporating measurements like bioimpedance analysis to assess lower extremity muscle strength more thoroughly in future investigations could provide additional insights. Lastly, it’s essential to recognize that various factors, including medication use and specific comorbid conditions, may have complex effects on these outcomes. Larger studies with a more comprehensive assessment of potential confounding variables may provide a more nuanced understanding of these relationships.

## 5 Conclusion

In summary, our study reveals noteworthy findings. Individuals with osteoporosis exhibit pronounced impairments in lower extremity proprioception compared to the control group. Additionally, individuals with osteoporosis display substantial compromises in postural stability, characterized by increased anterior–posterior sway, medial-lateral sway, and ellipse area compared to those without the condition. Furthermore, our investigation identifies significant negative correlations between proprioceptive function and postural stability measures within the osteoporosis group, emphasizing the crucial role of proprioception in maintaining postural control in this population. These findings carry significant clinical implications, highlighting the importance of tailored interventions addressing both bone health and proprioceptive deficits to enhance postural stability and reduce the risk of falls and fractures in individuals with osteoporosis.

## Data availability statement

The raw data supporting the conclusions of this article will be made available by the authors, without undue reservation.

## Ethics statement

The studies involving humans were approved by King Khalid University’s Research Ethics Committee. The studies were conducted in accordance with the local legislation and institutional requirements. The participants provided their written informed consent to participate in this study. Written informed consent was obtained from the individual(s) for the publication of any potentially identifiable images or data included in this article.

## Author contributions

IA: Conceptualization, Data curation, Formal analysis, Funding acquisition, Investigation, Methodology, Project administration, Resources, Software, Supervision, Writing – original draft, Writing – review & editing. RR: Conceptualization, Data curation, Formal analysis, Investigation, Methodology, Writing – original draft, Writing – review & editing. RA: Conceptualization, Data curation, Formal analysis, Investigation, Methodology, Writing – original draft, Writing – review & editing. JT: Conceptualization, Data curation, Formal analysis, Investigation, Methodology, Writing – original draft, Writing – review & editing. SD: Conceptualization, Data curation, Formal analysis, Writing – original draft, Writing – review & editing. HG: Conceptualization, Data curation, Formal analysis, Investigation, Methodology, Writing – original draft, Writing – review & editing. AA: Conceptualization, Data curation, Formal analysis, Investigation, Methodology, Writing – original draft, Writing – review & editing. SA: Writing – review & editing, Conceptualization, Data curation, Formal analysis, Investigation, Methodology, Writing – original draft. MJ: Conceptualization, Data curation, Formal analysis, Methodology, Writing – original draft, Writing – review & editing.

## References

[1] Bai R-J Li Y-S Zhang F-J. Osteopontin, a bridge links osteoarthritis and osteoporosis. Front Endocrinol. (2022) 13:1012508. doi: 10.3389/fendo.2022.1012508, PMID: 36387862 PMC9649917

[2] Dimai HP. New horizons: artificial intelligence tools for managing osteoporosis. J Clin Endocrinol Metabol. (2023) 108:775–83. doi: 10.1210/clinem/dgac702, PMID: 36477337 PMC9999362

[3] Yu P-A Hsu W-H Hsu W-B Kuo L-T Lin Z-R Shen W-J. The effects of high impact exercise intervention on bone mineral density, physical fitness, and quality of life in postmenopausal women with osteopenia: a retrospective cohort study. Medicine. (2019) 98:14898. doi: 10.1097/MD.0000000000014898 PMC6426501 30882707

[4] Li N. Osteoporosis, (bone) fractures and fracture liaison services: health-related quality of life, clinical and economic outcomes. Maastricht: Maastricht University (2023) 13:121.

[5] Lien W-C Ching CT-S Lai Z-W Wang H-MD Lin J-S Huang Y-C. Intelligent fall-risk assessment based on gait stability and symmetry among older adults using tri-axial accelerometry. Front Bioeng Biotechnol. (2022) 10:887269. doi: 10.3389/fbioe.2022.887269, PMID: 35646883 PMC9136169

[6] Gelener P İyigün G Özmanevra R. Proprioception and clinical correlation. Proprioception. IntechOpen. (2021) 2:95866. doi: 10.5772/intechopen.95866

[7] Reddy RS Tedla JS Dixit S Raizah A Al-Otaibi ML Gular K. Cervical joint position sense and its correlations with postural stability in subjects with fibromyalgia syndrome. Life. (2022) 12:1817. doi: 10.3390/life12111817, PMID: 36362972 PMC9697665

[8] Alhasan H Alshehri MA Wheele PC Fong DT. Effects of interactive videogames on postural control and risk of fall outcomes in frail and pre-frail older adults: a systematic review and meta-analysis. Games Health J. England: Loughborough University (2021). 10:83–94. 33651955 10.1089/g4h.2020.0009

[9] van Wieringen A Van Wilderode M Van Humbeeck N Krampe R. Coupling of sensorimotor and cognitive functions in middle-and late adulthood. Front Neurosci. (2022) 16:2042. doi: 10.3389/fnins.2022.1049639 PMC9752872 36532286

[10] Guillaud E Seyres P Barriere G Jecko V Bertrand SS Cazalets J-R. Locomotion and dynamic posture: neuro-evolutionary basis of bipedal gait. Neurophysiol Clin. (2020) 50:467–77. doi: 10.1016/j.neucli.2020.10.012, PMID: 33176989

[11] Akay T Murray AJ. Relative contribution of proprioceptive and vestibular sensory systems to locomotion: opportunities for discovery in the age of molecular science. Int J Mol Sci. (2021) 22:1467. doi: 10.3390/ijms22031467 33540567 PMC7867206

[12] El Miedany Y. Geroscience and management of osteoporosis in older adults. New horizons in osteoporosis. Management. (2022) 19:491–524. doi: 10.1007/978-3-030-87950-1_19

[13] Noamani A Lemay JF Musselman KE Rouhani H. Postural control strategy after incomplete spinal cord injury: effect of sensory inputs on trunk -leg movement coordination. J NEUROENG REHABIL. (2020) 17:1–2. 33109209 10.1186/s12984-020-00775-2 PMC7590439

[14] Wawrzyniak A Balawender K. Structural and metabolic changes in bone. Animals. (2022) 12:1946. doi: 10.3390/ani12151946, PMID: 35953935 PMC9367262

[15] Wang X Yang W Qin L. Prevention of osteoporotic fracture: from skeletal and non-skeletal perspectives. Frigid Zone Med. (2022) 2:214–24. doi: 10.2478/fzm-2022-0029

[16] Park S. Biochemical, structural and physical changes in aging human skin, and their relationship. Biogerontology (2022) 23:275–288. 35292918 10.1007/s10522-022-09959-w PMC10316705

[17] Tan MH. Editor. Diabetes mellitus: Impact on bone. Dental and Musculoskeletal Health Cambridge: Academic Press (2020) 132–168.

[18] Rogers ME Rogers NL Takeshima N Islam MM. Methods to assess and improve the physical parameters associated with fall risk in older adults. Prev Med. (2003) 36:255–64. doi: 10.1016/S0091-7435(02)00028-2 12634016

[19] Blauwet CA Brook EM Tenforde AS Broad E Hu CH Abdu-Glass E. Low energy availability, menstrual dysfunction, and low bone mineral density in individuals with a disability: implications for the para athlete population. Sports Med. (2017) 47:1697–708. doi: 10.1007/s40279-017-0696-0, PMID: 28213754

[20] Hulla R Gatchel RJ Liegey-Dougall A. Biopsychosocial measures related to chronic low back pain postural control in older adults. Healthcare. (2017) 5:74. doi: 10.3390/healthcare5040074 29036904 PMC5746708

[21] Corbeil P Simoneau M Rancourt D Tremblay A Teasdale N. Increased risk for falling associated with obesity: mathematical modeling of postural control. IEEE Trans Neural Syst Rehabil Eng. (2001) 9:126–36. doi: 10.1109/7333.928572, PMID: 11474965

[22] Rose DJ. Fallproof!: a comprehensive balance and mobility training program. Human Kinetics (2010) 14:172.

[23] Cuaya-Simbro G Perez-Sanpablo A-I Morales E-F Quiñones Uriostegui I Nuñez-Carrera L. Comparing machine learning methods to improve fall risk detection in older adults with osteoporosis from balance data. J Healthcare Eng. (2021) 2021:1–11. doi: 10.1155/2021/8697805, PMID: 34540190 PMC8448611

[24] Smith TO Davies L Hing CB. A systematic review to determine the reliability of knee joint position sense assessment measures. Knee. (2013) 20:162–9. doi: 10.1016/j.knee.2012.06.010, PMID: 22819143

[25] Lien W-C Guo N-W Chang J-H Lin Y-C Kuan T-S. Relationship of perceived environmental barriers and disability in community-dwelling older adults in Taiwan – a population-based study. BMC Geriatr. (2014) 14:1–8. doi: 10.1186/1471-2318-14-59 24885956 PMC4013536

[26] Relph N Herrington L Tyson S. The effects of ACL injury on knee proprioception: a meta-analysis. Physiotherapy. (2014) 100:187–95. doi: 10.1016/j.physio.2013.11.002, PMID: 24690442

[27] Clark NC Röijezon U Treleaven J. Proprioception in musculoskeletal rehabilitation. Part 2: clinical assessment and intervention. Man Ther. (2015) 20:378–87. doi: 10.1016/j.math.2015.01.009, PMID: 25787919

[28] Reddy RS Tedla JS Alshahrani MS Asiri F Kakaraparthi VN Samuel PS. Reliability of hip joint position sense tests using a clinically applicable measurement tool in older adults participants with unilateral hip osteoarthritis. Sci Rep. (2022) 12:376. doi: 10.1038/s41598-021-04288-3, PMID: 35013488 PMC8748869

[29] Saeed Alshahrani M Reddy RS Asiri F Tedla JS Alshahrani A Kandakurti PK. Correlation and comparison of quadriceps endurance and knee joint position sense in individuals with and without unilateral knee osteoarthritis. BMC Musculoskelet Disord. (2022) 23:444. doi: 10.1186/s12891-022-05403-9, PMID: 35549701 PMC9097169

[30] Alshahrani MS Reddy RS Tedla JS Asiri F Alshahrani A. Association between kinesiophobia and knee pain intensity, joint position sense, and functional performance in individuals with bilateral knee osteoarthritis. Healthcare. (2022) 10:120. doi: 10.3390/healthcare10010120 35052284 PMC8775958

[31] Alfaya FF Reddy RS Alshahrani MS Tedla JS Dixit S Gular K. Investigating the mediating role of pain in the relationship between ankle joint position sense and balance assessed using computerized posturography in individuals with unilateral chronic ankle instability: a cross-sectional study. Appl Sci. (2023) 13:8169. doi: 10.3390/app13148169

[32] Alshahrani MS Reddy RS. Relationship between kinesiophobia and ankle joint position sense and postural control in individuals with chronic ankle instability—a cross-sectional study. Int J Environ Res Public Health. (2022) 19:2792. doi: 10.3390/ijerph19052792, PMID: 35270483 PMC8910775

[33] Asiri F Reddy RS Alshahrani MS Tedla JS Dixit S Alshahrani A. Mediation effect of pain on the relationship between kinesiophobia and postural control: comparison and correlations in individuals with fibromyalgia syndrome and asymptomatic individuals—a cross-sectional study. Life. (2023) 13:175. doi: 10.3390/life13010175, PMID: 36676124 PMC9861203

[34] Kandakurti PK Reddy RS Kakarparthy VN Rengaramanujam K Tedla JS Dixit S. Comparison and association of neck extensor muscles’ endurance and postural function in subjects with and without chronic neck pain – a cross-sectional study. Physikalische Medizin, Rehabilitationsmedizin, Kurortmedizin. (2021) 31:295–301. doi: 10.1055/a-1395-1050

[35] Asiri F Reddy RS Narapureddy BR Raizah A. Comparisons and associations between hip-joint position sense and glycosylated hemoglobin in older adults subjects with type 2 diabetes mellitus—a cross-sectional study. Int J Environ Res Public Health. (2022) 19:15514. doi: 10.3390/ijerph192315514 36497588 PMC9741323

[36] Sedić A Pavković D Firak M. A methodology for normal distribution-based statistical characterization of long-term insolation by means of historical data. Sol Energy. (2015) 122:440–54. doi: 10.1016/j.solener.2015.09.014

[37] Goulet-Pelletier J-C Cousineau D. A review of effect sizes and their confidence intervals, part I: the Cohen’s d family. Q Methods Psychol. (2018) 14:242–65. doi: 10.20982/tqmp.14.4.p242

[38] Obilor EI Amadi EC. Test for significance of Pearson’s correlation coefficient. Int J Innov Math Stat Energy Pol. (2018) 6:11–23. doi: 10.3390/v14071504

[39] Pfeifer M Sinaki M Geusens P Boonen S Preisinger E Minne HW. Musculoskeletal rehabilitation in osteoporosis: a review. J Bone Miner Res. (2004) 19:1208–14. doi: 10.1359/JBMR.040507 15231006

[40] Correa RGP Silveira Gomes AR Borba VZC. Physiological and ankle functions are discriminating factors for the risk of falls in women in treatment of osteoporosis. Int J Environ Res Public Health. (2022) 19:12643. doi: 10.3390/ijerph191912643, PMID: 36231943 PMC9564876

[41] Ho PW Ma C. Falls prevention and osteoporotic fractures. The Routledge Handbook of Public Health and the Community (2021) 14:56.

[42] Angin S Simsek I. Comparative kinesiology of the human body: normal and pathological conditions Academic Press (2020) 66:74.

[43] Tornero-Aguilera JF Jimenez-Morcillo J Rubio-Zarapuz A Clemente-Suárez VJ. Central and peripheral fatigue in physical exercise explained: a narrative review. Int J Environ Res Public Health. (2022) 19:3909. doi: 10.3390/ijerph19073909, PMID: 35409591 PMC8997532

[44] O’Brien MS McDougall JJ. Age and frailty as risk factors for the development of osteoarthritis. Mech Ageing Dev. (2019) 180:21–8. doi: 10.1016/j.mad.2019.03.003, PMID: 30898635

[45] Sturnieks DL Tiedemann A Chapman K Munro B Murray SM Lord SR. Physiological risk factors for falls in older people with lower limb arthritis. J Rheumatol. (2004) 31:2272–9. 15517643

[46] Lorbergs AL MacIntyre NJ. The international classification of functioning, disability and health (ICF) core sets: application to a postmenopausal woman with rheumatoid arthritis and osteoporosis of the spine. Physiother Theory Pract. (2013) 29:547–61. doi: 10.3109/09593985.2013.773574, PMID: 23480536

[47] Pfeifer M Sinaki M Geusens P Boonen S Preisinger E Minne HW. ASBMR Working Group on Musculoskeletal Rehabilitation. Musculoskeletal rehabilitation in osteoporosis: a review. J Bone Miner Res. (2004) 19:1208–14. 15231006 10.1359/JBMR.040507

[48] Skelton DA Dinan-Young SM. Ageing and older people. Exercise physiology in special populations advances in sports and exercise science series. Philadelphia, USA: Churchill Livingstone (2008) 13:67.

[49] Okayama A Nakayama N Kashiwa K Horinouchi Y Fukusaki H Nakamura H. Prevalence of sarcopenia and its association with quality of life, postural stability, and past incidence of falls in postmenopausal women with osteoporosis: a cross-sectional study. Healthcare. (2022) 10:192. 35206807 10.3390/healthcare10020192 PMC8872599

[50] Simon A Rupp T Hoenig T Vettorazzi E Amling M Rolvien T. Evaluation of postural stability in patients screened for osteoporosis: a retrospective study of 1086 cases. Gait Posture. (2021) 88:304–10. doi: 10.1016/j.gaitpost.2021.06.013, PMID: 34166858

[51] Bartl R Frisch B. Osteoporosis: Diagnosis, prevention, therapy Springer Science & Business Media (2009) 23:39.

[52] Roya M Reza OG Azadeh S Reza HM Saeed TM. Changes of joint position sense in responses to upper trapezius muscle fatigue in subclinical myofascial pain syndrome participants versus healthy control. Muscles Ligaments Tendons J. (2018) 8:12. doi: 10.32098/mltj.04.2018.12

[53] Proske U Gandevia SC. The proprioceptive senses: their roles in signaling body shape, body position and movement, and muscle force. Physiol Rev. (2012) 92:1651–97. doi: 10.1152/physrev.00048.2011, PMID: 23073629

[54] Jerosch J Prymka M. Proprioception and joint stability. Knee surgery, sports traumatology, arthroscopy. (1996) 4:171–9. 10.1007/BF01577413 8961235

[55] Young WR Williams AM. How fear of falling can increase fall-risk in older adults: applying psychological theory to practical observations. Gait Posture. (2015) 41:7–12. doi: 10.1016/j.gaitpost.2014.09.006, PMID: 25278464

[56] Peterka RJ Loughlin PJ. Dynamic regulation of sensorimotor integration in human postural control. J Neurophysiol. (2004) 91:410–23. doi: 10.1152/jn.00516.2003, PMID: 13679407

[57] Burr DB. Bone morphology and organization. Basic and applied bone biology. Elsevier. (2019):3–26.

[58] Ferrucci L Baroni M Ranchelli A Lauretani F Maggio M Mecocci P. Interaction between bone and muscle in older persons with mobility limitations. Curr Pharm Des. (2014) 20:3178–97. doi: 10.2174/13816128113196660690, PMID: 24050165 PMC4586132

[59] Ucurum SG Altas EU Kaya DO. Comparison of the spinal characteristics, postural stability and quality of life in women with and without osteoporosis. J Orthop Sci. (2020) 25:960–5. doi: 10.1016/j.jos.2019.12.015, PMID: 32046937

[60] Rezaei MK Torkaman G Bahrami F Bayat N. Are weight shifting and dynamic control strategies different in postmenopausal women with and without type-I osteoporosis? Exp Gerontol. (2021) 154:111529. doi: 10.1016/j.exger.2021.111529, PMID: 34450234

[61] Macaulay TR Peters BT Wood SJ Clément GR Oddsson L Bloomberg JJ. Developing proprioceptive countermeasures to mitigate postural and locomotor control deficits after long-duration spaceflight. Front Syst Neurosci. (2021) 15:658985. doi: 10.3389/fnsys.2021.658985, PMID: 33986648 PMC8111171

[62] Efstathiou MA Giannaki CD Roupa Z Hadjisavvas S Stefanakis M. Evidence of distorted proprioception and postural control in studies of experimentally induced pain: a critical review of the literature. Scand J Pain. (2022) 22:445–56. doi: 10.1515/sjpain-2021-0205, PMID: 35470647

[63] Henry M Baudry S. Age-related changes in leg proprioception: implications for postural control. J Neurophysiol. (2019) 122:525–38. doi: 10.1152/jn.00067.2019, PMID: 31166819 PMC6734411

[64] Rathore R. Effect of age and fall status on the scaling of the postural control variables to a scaled gait initiation task Temple University (2020) 89:93.

[65] Hsu CL Nagamatsu LS Davis JC Liu-Ambrose T. Examining the relationship between specific cognitive processes and falls risk in older adults: a systematic review. Osteoporos Int. (2012) 23:2409–24. doi: 10.1007/s00198-012-1992-z, PMID: 22638707 PMC4476839

[66] Granacher U Gollhofer A Hortobágyi T Kressig RW Muehlbauer T. The importance of trunk muscle strength for balance, functional performance, and fall prevention in seniors: a systematic review. Sports Med. (2013) 43:627–41. doi: 10.1007/s40279-013-0041-1, PMID: 23568373

[67] Martin FC. Falls risk factors: assessment and management to prevent falls and fractures. Can J Aging/La Revue canadienne du vieillissement. (2011) 30:33–44. doi: 10.1017/S0714980810000747 24650637

[68] Ambrose AF Cruz L Paul G. Falls and fractures: a systematic approach to screening and prevention. Maturitas. (2015) 82:85–93. doi: 10.1016/j.maturitas.2015.06.035, PMID: 26255681

[69] Varahra A Rodrigues I MacDermid J Bryant D Birmingham T. Exercise to improve functional outcomes in persons with osteoporosis: a systematic review and meta-analysis. Osteoporos Int. (2018) 29:265–86. doi: 10.1007/s00198-017-4339-y, PMID: 29306984

[70] Madureira MM Takayama L Gallinaro A Caparbo V Costa R Pereira RM. Balance training program is highly effective in improving functional status and reducing the risk of falls in older adults women with osteoporosis: a randomized controlled trial. Osteoporos Int. (2007) 18:419–25. doi: 10.1007/s00198-006-0252-5, PMID: 17089080 PMC1820755

[71] Trevor SD. Resistance training for fall prevention: a narrative review of the literature. J Int Acad Neuromusculoskeletal Med. 19

[72] Reddy RS Alkhamis BA Kirmani JA Uddin S Ahamed WM Ahmad F. Age-related decline in cervical proprioception and its correlation with functional mobility and limits of stability assessed using computerized posturography: a cross-sectional study comparing older (65+ years) and younger adults. Healthcare. (2023) 11:1924. 37444758 10.3390/healthcare11131924 PMC10340456

[73] Gill-Body KM Hedman LD Plummer L Wolf L Hanke T Quinn L. Movement system diagnoses for balance dysfunction: recommendations from the academy of neurologic physical Therapy’s movement system task force. Phys Ther. (2021) 101:153. doi: 10.1093/ptj/pzab153 34160028

[74] Pinto D Alshahrani M Chapurlat R Chevalley T Dennison E Camargos B. The global approach to rehabilitation following an osteoporotic fragility fracture: a review of the rehabilitation working group of the international osteoporosis foundation (IOF) committee of scientific advisors. Osteoporos Int. (2022) 33:527–40. doi: 10.1007/s00198-021-06240-7, PMID: 35048200

